# Pain of Threatened Self: Explicit and Implicit Self-Esteem, Cortisol Responses to a Social Threat and Pain Perception

**DOI:** 10.3390/jcm13092705

**Published:** 2024-05-04

**Authors:** Ewa Wojtyna, Magdalena Hyla, Aleksandra Hachuła

**Affiliations:** 1 Institute of Medical Sciences, University of Opole, 45-040 Opole, Poland; 2 Institute of Psychology, University of Silesia in Katowice, 40-007 Katowice, Poland; magdalena.hyla@us.edu.pl; 3 Faculty of Psychology in Katowice, SWPS University, 40-326 Katowice, Poland; ahachula@st.swps.edu.pl

**Keywords:** self-esteem, cortisol response, pain, exclusion, trier social stress test

## Abstract

**Background**: Rejection, injustice, and exclusion from meaningful interpersonal relationships are often extremely painful and stress-generating experiences. This study aimed to define the role of explicit and implicit self-esteem in pain perception as a component of the physiological–psychological system that regulates the body’s response to stress associated with the threat of social rejection. **Methods**: In total, 360 individuals participated in this study. The measurement of cortisol in saliva, the assessment of pain thresholds using thermal stimuli, the IAT to assess implicit self-esteem, and a questionnaire on global self-esteem and social pain were used. The study included three measurements: baseline and 15 and 45 min after the application of a laboratory socially threatening stimulus (the Trier Social Stress Test). **Results**: People experiencing chronic social pain (CSP) are more likely to have fragile self-esteem, higher pain thresholds, and tend to experience reduced pain tolerance in situations of acute social threat than people without CSP experience. In people with CSP and fragile self-esteem, after the introduction of a social threat, an increase in pain tolerance was observed along with a longer-lasting increase in cortisol levels. **Conclusions**: Fragile self-esteem, along with feelings of chronic exclusion, injustice, and rejection, may prolong stress reactions and produce a hypoalgesic effect.

## 1. Introduction

Contemporary sociocultural changes expose people to many states in which their Self may be threatened. Numerous social comparisons, high demands, and expectations make it easy to become an object of unfair treatment or even exclusion, and individuals’ self-esteem can be painfully damaged [1,2]. The word *painfully* is not accidental here; all over the world, there are terms that describe difficult social situations in terms of physical tissue damage [3].

Pain, defined as “an unpleasant sensory and emotional experience associated with, or resembling that associated with, actual or potential tissue damage” [4], is one of the crucial challenges for interdisciplinary research and clinical teams. Several comments were added to the IASP definition of pain in 2020, clearly emphasizing that pain is a personal experience influenced by biological, psychological, and social factors and that people learn the concept of pain through life experiences. Among the various factors that may modulate the pain response, a social threat, understood as a situation in which one is exposed to negative social judgement and potential rejection by others, seems to deserve particular attention. While many previous studies have analyzed the relationships between stress and pain, self-esteem and pain, self-esteem and stress response, social threat and stress, and social pain and physical pain [3,5,6,7], there is still a lack of published evidence explaining the mechanism linking all these factors in a single model.

Rejection in meaningful interpersonal relationships is an extremely painful and anxiety-generating experience. Social threats trigger specific physiological changes [8,9]. Loosening interpersonal bonds or rejection by other group members is particularly risky. It requires implementing corrective measures because the sense of belonging to a social group is one of the basic needs of every human being. Evolutionary processes within the central nervous system result in the overlapping of neuronal circuits responsible for the reception of physical pain (nociception) with circuits processing information about a social threat [3,10,11,12]. Owing to the overlap of neuronal circuits, one may respond to social stimuli with physical pain. This phenomenon is known as social pain [3,10]. A social threat is likely associated with a specific physiological response involving activation of the nervous, endocrine, and immune systems.

The primary response to stress is the activation of a neural pathway within the sympathetic nervous system, which results in the release of catecholamines. Within approximately 30 min of exposure to a stressor, the hormonal system regulating the stress response is activated; this system is linked to the hypothalamic-pituitary-adrenal axis (HPA) and the release of glucocorticoids, especially cortisol [8].

In their theory of social self-preservation, Dickerson et al. [11] postulated that a social threat to the self, including a threat to self-esteem, position in a group, and acceptance, triggers a specific pattern of biopsychological response that includes the activation of the HPA axis. Furthermore, a meta-analysis by Dickerson and Kemeny [8] demonstrated that social threat evokes the most potent physiological, endocrine, and immune responses to all equally intensive stressors. These observations were confirmed by the results of further studies, in which the increase in cortisol secretion turned out to be particularly evident during tasks associated with social judgement [9,13,14,15]. While such a response has an adaptive function during exposure to an acute social threat, a prolonged stress response may also have some negative health consequences, such as cardiovascular complications, psychosomatic disorders, impairment of immunity, or enhanced inflammatory and oxidative reactions leading to cellular damage [16,17]. However, the mechanism underlying the prolonged stress response to an acute social threat is still poorly understood. Although Dickerson and Kemeny [8] already postulated that some individual traits might modulate stress responses, there is still a paucity of empirical data on these factors [18,19]. Self-esteem may be one such factor. The expansive culture of the West is based on individualism; therefore, there is a constant increase in the importance of the status and importance of the individual [20]. Self-esteem is the same factor that is worth looking at with special attention. The Self does not function in isolation; relationships with other people are important for building self-esteem [21]. In this context, an individual’s previous experiences of rejection or unfair treatment by the social environment also become important.

Self-esteem may determine one’s threshold of sensitivity to rejection signaling. Individuals with low self-esteem frequently strive for a permanent sense of being valuable and try to verify whether others genuinely take care of them [22]. In line with the model of risk regulation in close interpersonal relationships, individuals with low self-esteem show self-protective mechanisms, such as early detection of rejection signaling, interpretation of ambiguous signals as threatening, or avoiding confrontation with potential rejection [23,24,25,26]. In contrast, people with high self-esteem may be more resistant to rejection owing to a lesser need for a protective decrease in the sensitivity thresholds for threatened rejection [22]. Ford and Collins [5] demonstrated that individuals with high self-esteem show a weaker cortisol response to experimentally induced rejection (failure in online dating) than those with low self-esteem.

However, self-esteem may vary depending on the situational context. Therefore, state and trait self-esteem estimates may differ [27,28]. Kernis and Waschull [28] emphasized that subjects with unstable self-esteem over time have a stronger tendency to have self-protective attitudes than those with stable self-esteem. Seery et al. [29] demonstrated that when experiencing failure, subjects with unstable high self-esteem showed a physiological response typical for a threat, whereas subjects with stable high self-esteem responded to it as a challenge, although both reactions were associated with exacerbating the sympathetic stress response. However, the same study revealed that individuals with unstable high self-esteem also showed a change in the parameters of the cortisol-dependent stress response. This finding implies that unstable self-esteem may be associated with a prolonged stress response.

In the abovementioned studies, self-esteem was estimated using a self-inventory (all results refer to explicit self-esteem). However, measures of explicit self-esteem (ESE), understood as a global subjective self-assessment of one’s value [21,30], are prone to numerous confounders and distortions, such as a tendency to overestimate one’s value, self-deception, or the need for social approval [31]. In addition to explicit self-esteem, psychology distinguishes another aspect of this factor, the so-called implicit self-esteem. Implicit self-esteem (ISE) is defined as the introspectively unidentified effect of self-attitude on the evaluation of self-associated and self-dissociated objects [32].

ESE is linked to a cognitive system, whereas ISE is linked to an experience-based system [30]. ISE develops as the first primitive trait and is tightly bound to childhood experiences [33]. The discrepancy between ESE and ISE is a measure of self-esteem stability [34]. Such an approach to self-esteem stability seems to be particularly valuable, since persons who explicitly declare high self-esteem may constitute a heterogeneous group, including both subjects with secure high self-esteem (i.e., high ESE and ISE) and individuals with fragile self-esteem (i.e., high ESE and low ISE) [34,35,36,37].

Secure self-esteem is defined as a well-rooted sense of self-value based on a realistic self-image that is difficult to undermine. In turn, fragile self-esteem is described as a non-realistic sense of self-esteem that is susceptible to harm and must constantly be confirmed. Individuals with low implicit self-esteem more frequently determine their value based on external indices such as image, academic accomplishments, approval from others, and competition [37]. Rumination about the need to maintain self-esteem from a longer perspective may also be associated with prolonged stress response in such individuals. In the event of social comparison, the mechanism of self-esteem compensation may mobilize a greater effort to catch up with others.

Individuals exposed to chronic stress may be more prone to anticipation stress and develop a form of strong stress response characterized by the preterm release of cortisol [38,39,40]. Presumably, people with a strong need to confirm their high position in a social group are permanently exposed to chronic stress. Chronic experiences of stigmatization and social exclusion are frequently shown to predispose individuals to low self-esteem [41,42] and, consequently, may also trigger self-esteem hyper-compensation.

Chronic stress may lead to greater sensitivity to pain based on neurobiological mechanisms. The stress response and the response to pain involve similar neuronal and hormonal circuits. Glucocorticoids attenuate the pain response and cause an increase in the pain threshold due to the activation of glucocorticoid receptors and the resultant release of beta-endorphins from the pituitary gland [43]. While the activation of the HPA axis is beneficial in the case of acute stress, its function can be impaired by chronic stress, which results in excessive or insufficient release of cortisol, impairment of cortisol reactivity, or disruption of circadian release [44,45]. Such dysregulation of the HPA axis is, in turn, associated with greater pain severity [46]. Persons with a flattened cortisol awakening response (CAR; an increase in cortisol within the first hour after awakening that is separate from the cortisol increase during the second half of the night) experienced more severe, hardly bearable pain after thermal stimulation during the cold pressor test [47]. Furthermore, prolonged stress response has been shown to be associated with enhanced oxidative stress [17], and as such, may be an essential modifier of pain and inflammatory response.

Self-esteem has been shown to be linked to the activity of some specific brain areas that determine the sense of social distress and pain [48,49,50,51]. According to Somerville et al. [51], negative feedback regarding social relationships is associated with anterior cingulate cortex (ACC) activation. In the studies conducted by these authors, individuals with low self-esteem showed stronger ACC activation. Stronger activation of the ACC and anterior insula in response to rejection signaling has also been documented in subjects with greater susceptibility to rejection [52,53]. Importantly, activation of this CNS area is also often associated with distress caused by physical pain [53,54] or distress related to a social threat [48,50,54,55,56].

Altogether, the evidence above implies that people exposed to a social threat may also be more sensitive to pain. In contrast, a series of experiments conducted by DeWall and Baumeister [57] demonstrated that a sense of social exclusion contributes to an increase in the pain threshold and pain tolerance. These discrepancies can be interpreted, in part, in the context of safe and fragile self-esteem.

The type of physiological stress response determines pain tolerance; however, the exact underlying mechanisms of this relationship are still not understood. This relationship may be mediated by other variables, including psychosocial factors (such as self-esteem and chronic social stress). The current study is the first to analyze the stress response and changes in pain tolerance of people with various patterns of explicit and implicit self-esteem in a single model.

This study aimed to define the role of explicit and implicit self-esteem in pain perception and tolerance as a component of the physiological-psychological system that regulates the body’s response to stress associated with a threat of social rejection.

The following hypotheses were formulated: (1) Individuals with fragile self-esteem (high explicit self-esteem and low implicit self-esteem) will demonstrate higher pain tolerance in response to a socially threatening situation compared to individuals with secure self-esteem (high explicit and implicit self-esteem or low–high explicit and implicit self-esteem); (2) Individuals with fragile self-esteem will exhibit a stronger stress reaction, evidenced by a greater increase in cortisol levels, in response to a social threat compared to those with secure self-esteem; (3) Individuals with chronic social pain will demonstrate lower pain tolerance in response to a socially threatening situation compared to individuals without experience of chronic social pain; (4) The increase in cortisol levels following the introduction of a social threat will be more pronounced among individuals experiencing chronic social pain than those without such experiences; (5) Individuals experiencing chronic social pain will exhibit a more prolonged cortisol response to social threat situations compared to those without chronic social pain; (6) The response to a social threat will vary depending on whether the threat is posed by potentially friendly or hostile jurors.

## 2. Materials and Methods

### 2.1. Participants

The recruitment of study participants took place via leaflets and information posted on social media and websites, including information regarding minorities particularly vulnerable to chronic social pain (e.g., people from LGB groups). The inclusion criteria for the study were age 18–40, professional or school activity, BMI in the range of 18.5–24.9 and consent to participate in the study. Exclusion criteria were the absence of endocrine, neuropsychiatric, and metabolic disorders; the use of steroid hormones, psychoactive substances, and other substances that may affect cortisol levels; and shift work.

**Figure 1:**
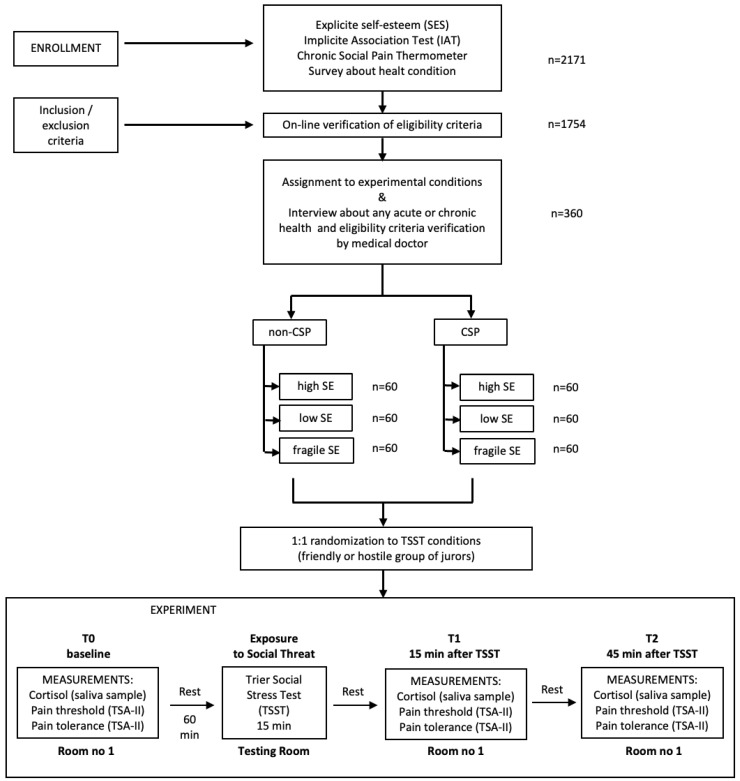
Research study flowchart. Note: CSP—chronic social pain; SE—self-esteem; TSST—Trier Social Stress Test.

Of the 2171 individuals who registered for the study and completed a set of preliminary tests (allowing for the assessment of explicit self-esteem, implicit self-esteem, and chronic social pain), 1754 met the eligibility criteria. These individuals were classified by explicit and implicit self-esteem profiles and the presence or absence of chronic exposure to social pain according to the main conditions assumed in the study (Figure 1). The study considered the following conditions: (a) presence or absence of a sense of chronic social pain and (b) low, high, or fragile self-esteem. To participate in the appropriate part of the study, 60 people who met the criteria for a given condition were randomly selected for each experimental condition. In the final stage, each of the selected subgroups was randomly divided into the following two experimental conditions related to the experimental manipulation in the Trier Social Stress Test: (c1) threat of social rejection by jurors from a potentially friendly group to the subject, and (c2) threat of social rejection by jurors from a potentially hostile group.

Thus, 12 subgroups of 30 people were ultimately selected to participate in the experiment. The descriptive characteristics of the study participants are presented in Table 1.

### 2.2. Study Design

All invited participants completed three stages of qualification for the main study. In the first stage, applicants completed the Rosenberg Self-Esteem Scale (SES), Implicit Association Test (IAT), Chronic Social Pain Thermometer, and a survey on socioeconomic status, health problems, and health behaviors (Figure 1). Candidates for the main study were selected and invited to an online interview, during which a clinical interview was conducted to determine compliance with the inclusion and exclusion criteria of the study. In the third stage of qualification, candidates were invited to the laboratory, where the medical doctor assessed compliance with the inclusion and exclusion criteria and the correctness of preparation for the examination (candidates received written information with instructions on how to prepare for the laboratory session).

Laboratory examinations were performed between 10:00 a.m. and 1:00 p.m. The subjects were invited to room no. 1, where they collected saliva samples and determined the pain and pain tolerance thresholds. After 60 min, during which the study participants rested, they were invited to the testing room, where the TSST was administered for 15 min. After completing the TSST, the subjects returned to room no. 1, where 15 and 45 min after completing the TSST, saliva samples were collected again, and pain thresholds and pain tolerance were measured.

After completing the study, participants were provided with information and answers to all questions. The subjects received reimbursement of PLN 100 (approximately USD 25) for their time and travel expenses. The Research Ethics Committee of the Institute of Psychology of the University of Silesia in Katowice approved this study.

### 2.3. Methods

Salivary cortisol concentration was used to measure the stress reaction. Saliva samples (1–1.5 mL) were collected via passive salivation. The samples were frozen and stored at −20 °C for further analysis. The study used Cortisol Saliva enzyme immunoassay kits (DiaMetra, Spello-Perugia, Italy; sensitivity: 0.12 ng/mL; standard range: 0.5–100 ng/mL).

Pain threshold and tolerance were estimated based on responses to warm thermal stimuli. The thermal stimuli were generated using a Peltier-based thermode with a 30 × 30 mm surface of the thermo-sensory stimulator (TSA-II; Medoc, Ramat Yishay, Israel). The temperature of the thermode will increase continuously from 30 °C to 50.5 °C at a rate of 1.5 °C/s. The lowest temperature at which the subject reported the first sensation of pain and the temperature at which he/she discontinued the experiment due to the presence of unbearable pain were recorded as the pain threshold and pain tolerance level, respectively.

The Rosenberg Self-Esteem Scale (SES) is a 10-item instrument commonly used to assess overall self-esteem [58,59]. Examples of items included in the tool are “On the whole, I am satisfied with myself” or “All in all, I am inclined to feel that I am a failure.” The SES has been used in many previous studies of explicit and implicit self-esteem. A higher score indicates higher explicit self-esteem. The Cronbach’s alpha reliability coefficient for the Polish version of the SES is 0.82 [59].

The Chronic Social Pain Thermometer [6], the original instrument designed for this study, is a visual analogue scale (0–100) resembling a commonly used pain severity scale. Participants were asked to assess their feelings on three thermometric scales: (1) social exclusion, (2) unfair treatment, and (3) a sense of loss in meaningful social relationships. Participants were asked to provide ratings for the past 12 months. A similar procedure, referring to the feeling of loss, was already used in our previous research [6]. Using this instrument enabled the identification of entities experiencing various types of chronic social pain: loss, exclusion, or injustice. The overall result, that is, the sense of chronic social pain, was calculated as the arithmetic mean of the results obtained for the three dimensions examined: social exclusion, unfair treatment, and loss in meaningful social relationships. The higher the score, the stronger was the feeling of social pain. The Cronbach’s alpha reliability coefficient for the total score is 0.81. Individuals who scored at least 67 points (the top third of the scale) were categorized as having chronic social pain. People who scored up to 33 points were categorized as those without a sense of chronic social pain.

The Implicit Association Test (IAT) is one of the most common methods for determining hidden self-esteem [60]. During the IAT, the subject is asked to classify stimuli belonging to the so-called target categories “ME” and “NOT-ME” to one of two affective categories (“positive”/“negative”). In other words, it should correctly classify the word displayed in the centre of the screen (e.g., “my face”) into one of two affective categories displayed in the upper left and right corners of the screen (e.g., “happiness,” “illness”) in the shortest possible time. Categorization was performed by hitting a designated key on a computer keyboard [60]. The scoring is based on the IAT d parameter [60]. Positive values of the d parameter indicate high implicit self-esteem, whereas negative values indicate low implicit self-esteem. Based on the IAT test results and SES results, the subjects were classified as those with fragile high self-esteem (high explicit and low implicit self-esteem), secure low self-esteem (low explicit and low implicit self-esteem), and secure high self-esteem (high explicit and high implicit self-esteem).

The Trier Social Stress Task (TSST) is a conventional procedure used to determine a subject’s response to a social threat [61]. During the TSST procedure, respondents had five minutes to prepare a statement about themselves, presenting themselves as the optimal candidate for a position they considered attractive. They then had five minutes to make a speech. After completing this task, the subjects were asked to solve an arithmetic problem (counting backward from 2013 in steps of 17) within the shortest possible time. While performing the task, the subjects were informed of the remaining time using an acoustic signal [62]. During the TSST, participants were observed by a group of five jurors, all of whom showed clear signs of increasing boredom. Before introducing the TSST, the study participants were randomly assigned to one of two experimental conditions, differing in information about jurors’ attitudes towards the participant (friendly attitude vs. hostile). In one case, the subjects received signals that the jurors belonged to the same or similar social group as the subjects (friendly condition). In the second condition, the subjects interacted with jurors who were members of a potentially competitive social group (hostile condition). During the TSST, the participants were observed in each experimental condition by a group of five jurors, all of whom showed clear signs of increasing boredom.

### 2.4. Data Analysis

Statistical data analysis was performed using the SPSS v28.0.1.1 statistical package (IBM Corp., Armonk, NY, USA). Normal distribution was tested using the Kolmogorov–Smirnov test. If the data were not normally distributed, they were transformed using log transformation. Cortisol data were centered and log-transformed to reduce skewness. A repeated-measurement analysis of variance with “group” and “time” was performed to investigate the effect of social threat on cortisol response and pain perception. Bonferroni-corrected post hoc tests were performed as required. For all the above analyses, *p*-values of less than 0.05 were considered significant.

## 3. Results

In the group of people experiencing chronic social pain (from now on CSP) compared to people who did not declare such experience (from now on non-CSP), there were significantly more people with non-heterosexual orientation, fewer people with higher education, and fewer people living in larger cities. A lower average level of global self-esteem and a higher percentage of fragile self-esteem were found in the CSP group (Table 1).

**Figure 2:**
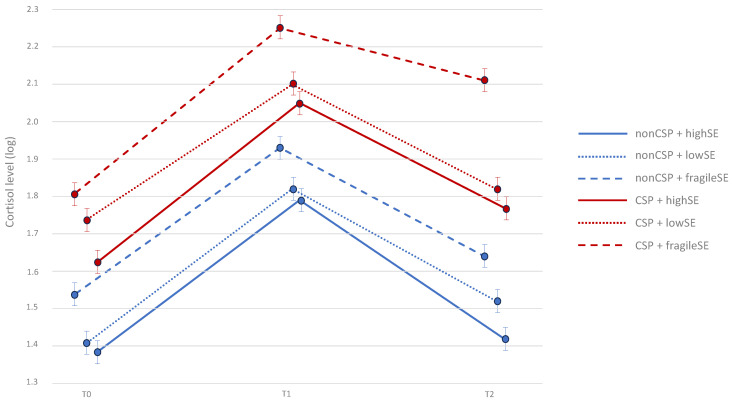
Cortisol levels at baseline (T0) and 15 min (T1) and 45 min (T2) after social threat situation (TSST); CSP—chronic social pain; SE—self-esteem.

People with fragile self-esteem showed the highest cortisol level among all the subjects before the introduction of the social threat situation (Trier Social Stress Test, TSST) and 15 and 45 min after the introduction of the TSST (*p* < 0.001) (Figure 2). People from the CSP group showed significantly higher salivary cortisol levels at baseline and at 15 and 45 min after the TSST than those from the non-CSP group (*F* = 18.02; *p* < 0.001). At 45 min after completing the TSST, compared with the results at 15 min after completing the TSST, a smaller decrease in cortisol levels was observed in the CSP group than in the non-CSP group (*p* = 0.041). The smallest decrease in cortisol levels between 15 and 45 min after the TSST was observed in people from the CSP group who underwent the TSST under the threat of rejection from their own (potentially friendly) social group (*F* = 6.42; *p* = 0.004). This cortisol level decrease was significantly smaller than the decrease in cortisol levels between measurements from 15 to 45 min after TSST in people from the CSP group who experienced rejection signals from people from potentially hostile (*F* = 4.53, *p* = 0.010) and friendly (*F* = 3.13; *p* = 0.031) social groups in the TSST experiment.

**Figure 3:**
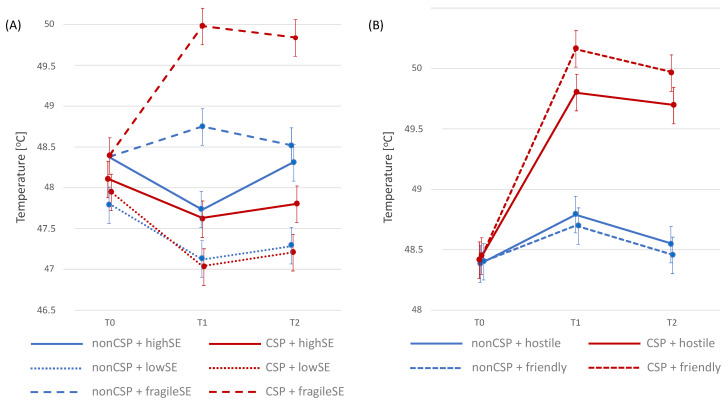
Pain tolerance at baseline (T0) and 15 min (T1) and 45 min (T2) after social threat situation (TSST); (**A**) according to chronic social pain (CSP) and self-esteem (SE); (**B**) among participants with fragile self-esteem according to the hostile or friendly condition of TSST.

The baseline pain thresholds were the highest in CSP individuals with fragile self-esteem and the lowest in those with low self-esteem (Table 2, Figure 3A).

After the social threat situation (TSST) was introduced, the non-CSP group with secure high self-esteem showed the most significant change in pain tolerance threshold (decrease in pain tolerance; *F* = 38.02; *p* < 0.001). However, 45 min after completing the TSST, the pain tolerance threshold in this subgroup increased again to the baseline level (*p* = 0.897). In the CSP group with fragile self-esteem, an increase in pain thresholds (*F* = 3.14; *p* = 0.389) and pain tolerance (*F* = 39.10; *p* < 0.001) was observed 15 min after the introduction of a social threat. In the case of pain tolerance, this change persisted also for 45 min after the TSST. The most significant increase in the pain tolerance threshold was noted in people from the CSP group with fragile self-esteem who experienced rejection by their own social group during the TSST (*F* = 24.65; *p* < 0.001; Figure 3B).

A positive correlation was observed between the pain tolerance threshold 45 min after TSST and cortisol levels (*tau* = 0.38; *p* < 0.01).

## 4. Discussion

Our study aimed to expand current knowledge on the biological effects of social pain by examining the cortisol response to social threat stress and pain sensitivity. We asked the research question of whether the chronic experience of social pain and the type of self-esteem differentiate the biological response to the threat of rejection. The hypotheses were confirmed. The results of the experiment show that there are significant differences between people with and without chronic stress and between people with secure and fragile self-esteem.

In our study, we observed higher cortisol levels in people with experience of CSP compared to people without CSP, both before and after exposure to the acute threat of social rejection, especially in people with fragile self-esteem. However, the pain response observed in our study followed a different pattern. People with secure self-esteem with and without comorbid experiences of chronic social pain reacted according to assumptions about the hyperalgesic effect of stimuli related to social threat [63]. This finding was consistent with the concept of social pain [3,6,10,12]. However, a significant increase in pain tolerance was observed in people with fragile self-esteem and CSP. The hypoalgesic responses to pain and increased HPA axis reactivity in these individuals are reminiscent of the responses observed in some PTSD studies. Similar to Defrin et al. [64], Hood and Badour [65] showed increased pain thresholds and pain tolerance in people with severe PTSD symptoms, suggesting a hypoalgesic response to pain. Many years of experiencing injustice, exclusion, or loss in relationships (social pain) can be treated as a situation similar to the phenomena observed in the case of complex trauma [66].

Our results also indicate prolonged (45 min after a social threat) elevated cortisol levels in people with CSP and fragile self-esteem, especially in conditions when rejection is generated by one’s own potentially friendlier social group. Stimuli suggesting exclusion or unfair treatment may appear very often for people from this subgroup. Thus, their reactivity to stress may be reduced owing to chronic hypercortisolemia. This finding is consistent with the observations of Aschbacher et al. [38]. In a study determining the pain tolerance threshold during the cold pressor test, lesser circadian variability in cortisol release was associated with a greater severity of tolerated pain [67]. This implies that individuals with such a pattern of cortisol release may bear stronger pain. Presumably, a mechanism similar to that identified in the case of chronic stress and anticipation stress response may be involved; fragile self-esteem and/or a chronic sense of social exclusion may result in the development of a protective mechanism against chronic discomfort associated with pain [57].

Moreover, individuals with fragile high self-esteem were shown to be more defensive than individuals with high ESE and ISE [68,69] and may show more negative affect, anger, hostility, or aggression, especially when their positive self-image is threatened [34,68,70]. Moreover, persons with high ESE and low ISE are less likely to forgive and more often declare the desire to take revenge on their offenders, especially if the latter apologizes to them since they consider an apology as a blame confession [71]. Rumination about revenge or the need to maintain self-esteem from a longer perspective may also be associated with prolonged stress responses in such persons.

In our study, a large proportion of the participants in the CSP group were non-heterosexual. There is a common feeling of injustice and exclusion in this social group, and so-called minority stress is an additional burden on these people. Mijas et al. [72] reported significantly higher cortisol levels during exposure to a socially threatening stressor, predicted by minority stressors.

Our study has several limitations. The sizes of the analyzed subgroups were relatively small, which made it impossible to control for numerous confounding factors. The participants included people from a minority group with a non-heterosexual orientation. However, little is known about whether participants included representatives from other minority groups. Thus, the diversity of the subjects remains implicit. In this study, we considered the subjective feeling of being excluded or treated unfairly when assessing social pain. However, we do not know what may have caused this feeling. More attention should be paid to this aspect in future studies. Many factors influence the stress and pain response, which we could not control in this study. However, the obtained results indicate the usefulness of considering the fragility of self-esteem in future research. It is also worth examining other ways to measure cortisol levels. Saliva samples were used in this study. This measurement depends on several factors that are difficult to control. Perhaps extending the research to measure cortisol in hair samples [63], enabling the assessment of cortisol levels over the last three months, would provide additional value in analyzing the relationships between stress, pain, and social threat.

Future research should pay attention to factors that may mediate or moderate the observed relationships between social threat, self-esteem, social pain, and stress reactions. Perhaps the introduction of personality factors into the model, especially neuroticism [73,74], resilience [75,76], self-compassion [77], and self-stigma [78], will allow us to observe new paths in understanding the reaction to social threats.

The results of our study have implications for clinical practice. We point out the particular need to care for people with fragile self-esteem who experience the feeling of being rejected or treated unfairly in their everyday lives. Building stable self-esteem requires introducing specific psychotherapeutic methods, including improving psychological flexibility [79,80] and emotional schema therapy [81]. Among all the participants in our study, these individuals were also most likely to experience a stronger and prolonged stress reaction after a social threat. This means that these people may be more likely to develop stress-related conditions that impair well-being and may be too late to seek help. This delay may be related to the increase in pain tolerance after the social threat observed in the study.

## 5. Conclusions

People experiencing chronic social pain are more likely to have fragile self-esteem, higher pain thresholds, and tend to experience reduced pain tolerance in situations of acute social threat than people without experience of chronic social pain. The threat of social rejection is a stimulus that increases cortisol reaction and sensitivity to pain. However, the course of this reaction is related to the fragility of the participants’ self-esteem. When experiencing rejection, pain sensitivity decreases, except for people with fragile self-esteem, whose pain tolerance increases, especially in people experiencing chronic social pain.

## Figures and Tables

**Table 1:** Descriptive characteristics of study participants.

Characteristics	CSP Group (*n* = 180)	Non-CSP Group (*n* = 180)	*p*-Value
Age [*M* (*SD*)]	28.16 (4.30)	27.82 (5.03)	0.918
Gender [*n* (%)]			
men	82 (45.6)	83 (46.1)	0.964
women	94 (52.2)	97 (53.9)	0.659
others	4 (2.2)	-	-
Psychosexual orientation [*n* (%)]			
Non-heterosexual	121 (67.2)	46 (25.6)	<0.001
Education [*n* (%)]			
primary	8 (4.5)	6 (3.3)	0.562
vocational	36 (20.0)	33 (18.3)	0.801
secondary	60 (33.3)	52 (28.9)	0.047
higher	76 (42.2)	89 (49.5)	0.035
Place of residence			
city size > 100 k [*n* (%)]	141 (78.3)	150 (83.3)	0.042
Self-esteem			
global (explicit) [*M* (*SD*)]	28.72 (8.95)	34.82 (6.18)	0.026
high [*n* (%)]	48 (26.7)	70 (38.9)	<0.001
low [*n* (%)]	31 (17.2)	32 (17.8)	0.949
fragile [*n* (%)]	101 (56.1)	78 (43.3)	<0.001
Cortisol level (nmol/L) [median (IQR)]			
T0 (baseline)	16.47 (15.48)	12.62 (10.11)	0.012
T1 (15 min after TSST)	24.59 (21.83)	17.45 (20.34)	<0.001
T2 (45 min after TSST)	18.25 (13.27)	14.21 (12.08))	0.009

Note: CSP—chronic social pain.

**Table 2:** The thresholds of pain and pain tolerance according to chronic social pain and self-esteem.

Group	Self-Esteem	Pain Threshold (T0)	Pain Tolerance		
			T0	T1	T2
non-CSP	low	45.82 (4.18)	47.80 (4.24)	47.12 (5.07)	47.29 (4.87)
	high	46.47 (4.29)	48.37 (4.41)	47.74 (5.22)	48.32 (5.04)
	fragile	46.86 (5.06)	48.38 (4.45)	48.75 (5.87)	48.51 (5.43)
CSP	low	45.08 (5.26)	47.96 (4,12)	47.04 (5.12)	47.21 (5.01)
	high	46.73 (5.01)	48.10 (4.09)	47.63 (5.04)	47.80 (4.92)
	fragile	46.94 (6.12)	48.40 (5.08)	49.98 (5.18)	49.84 (5.09)

Note: CSP—chronic social pain; n_non-CSP_ = 180; n_CSP_ = 180.

## Data Availability

The data presented in this study are available on request from the corresponding author.
